# Supplementary material for: Reconciling Mining with the Conservation of Cave Biodiversity: A Quantitative Baseline to Help Establish Conservation Priorities
Source: PLoS One. 2016 Dec 20;11(12):e0168348. doi: 10.1371/journal.pone.0168348 (PMC5173368; doi:10.1371/journal.pone.0168348)
Supplement: S1 Dataset — (ZIP) [file pone.0168348.s002.zip › Taxa/Serra Norte/SN_2013/N4WS/Lista N4WS-72.pdf]

## CAVIDADE N4WS-0072

| Classe | Ordem | Fam/Outros | Gên/Outros | Espécie | S-U | U-S | Zon. |
|---|---|---|---|---|---|---|---|
| Annelida | Oligochaeta | | | sp.1 | 31 | 1 | P |
| Arachnida | Acari | (Oribatida) | | sp.1 | X | | P |
| Arachnida | Acari | (Oribatida) | | sp.3 | | X | |
| Arachnida | Acari | (Oribatida) | | sp.4 | | X | A |
| Arachnida | Acari | (Parasitengonina) | | sp.3 | | X | A |
| Arachnida | Acari | (Parasitengonina) | | sp.5 | X | | A |
| Arachnida | Acari | (Uropodina) | | sp.2 | | X | E |
| Arachnida | Acari | (Uropodina) | | sp.2 | X | | E/P |
| Arachnida | Acari | (Uropodina) | | sp.3 | | X | P |
| Arachnida | Acari | (Uropodina) | | sp.3 | X | | P |
| Arachnida | Acari | Acaridae | | sp.1 | | X | P |
| Arachnida | Acari | Ameroseiidae | | sp.1 | | X | |
| Arachnida | Acari | Anoetidae | | sp.1 | | X | P/A |
| Arachnida | Acari | Ascidae | | sp.1 | X | | P |
| Arachnida | Acari | Laelapidae | *Cosmolaelaps* | sp.1 | | X | P |
| Arachnida | Acari | Macrochelidae | | sp.1 | | X | E/A |
| Arachnida | Acari | Macronyssidae | | sp.1 | X | | P |
| Arachnida | Acari | Mesostigmata | | sp.1 | X | X | P/A |
| Arachnida | Acari | Mesostigmata | | sp.2 | X | | P |
| Arachnida | Acari | Trombiculidae | | sp.1 | X | | P |
| Arachnida | Acari | Tydeidae | | sp.1 | | X | |
| Arachnida | Amblypygi | Phrynidae | *Heterophrynus* | *longicornis* | 17 | 7 | P/A |
| Arachnida | Araneae | Corinnidae | *Creugas* | sp.1 | 15 | 14 | E/P/A |
| Arachnida | Araneae | Ctenidae | *Nothroctenus* | *marschi* | 2 | 4 | P |
| Arachnida | Araneae | Filistatidae | | jovem | X | X | E |
| Arachnida | Araneae | Ochyroceratidae | *Speocera* | sp.1 | X | | P |
| Arachnida | Araneae | Pholcidae | *Leptopholcus* | sp.1 | X | | E |
| Arachnida | Araneae | Pholcidae | *Mesabolivar* | *eberhard* | X | X | P/A |
| Arachnida | Araneae | Scytodidae | *Scytodes* | *eleonorae* | 3 | 1 | P |
| Arachnida | Araneae | Theraphosidae | aff. *Holothele* | sp.1 | 11 | 20 | E/P |
| Arachnida | Araneae | Theridiidae | *Achaearanea* | sp.1 | X | X | P/A |
| Arachnida | Pseudoscorpiones | Chernetidae | *Spelaeochernes* | sp.1 | X | X | E/P |
| Arachnida | Pseudoscorpiones | Chthoniidae | | sp.4 | | X | P |
| Arachnida | Schizomida | Hubbardiidae | aff. *Rowlandius* | sp.1 | | X | P |
| Entognatha | Collembola | Cyphoderidae | | sp.1 | X | X | E/P |
| Entognatha | Collembola | Paronellidae | | sp.1 | | X | P |
| Entognatha | Collembola | Paronellidae | | sp.4 | X | X | P/A |
| Entognatha | Collembola | Symphypleona | | sp.1 | X | X | E |
| Insecta | Blattodea | Blaberidae | | sp.1 | 3 | | P |
| Insecta | Blattodea | Blaberidae | | sp.1 e sp.2 | 34 | 94 | P |
| Insecta | Blattodea | Blaberidae | | sp.2 | 2 | | P |
| Insecta | Blattodea | Blattellidae | | sp.1 | 16 | 1 | P |
| Insecta | Blattodea | Blattidae | | jovem | | 3 | E/P |
| Insecta | Coleoptera | Curculionidae | Platypodinae | sp.3 | X | | P |
| Insecta | Coleoptera | Histeridae | | sp.1 | X | | P |
| Insecta | Coleoptera | Histeridae | | sp.2 | X | X | P/A |
| Insecta | Coleoptera | Histeridae | | sp.3 | X | | P |
| Insecta | Coleoptera | Hydrophilidae | Sphaeridiinae | sp.4 | X | X | P/A |
| Insecta | Coleoptera | Ptiliidae | | sp.1 | | X | |

| | | | | | | | | |
|---|---|---|---|---|---|---|---|---|
| Insecta | Coleoptera | Scarabaeidae | | sp.1 | | X | P/A |
| Insecta | Coleoptera | Staphylinidae | Pselaphinae | sp.34 | | X | P |
| Insecta | Coleoptera | Staphylinidae | | sp.35 | | X | A |
| Insecta | Coleoptera | Staphylinidae | | sp.40 | X | X | P/A |
| Insecta | Coleoptera | Staphylinidae | | sp.66 | | X | E |
| Insecta | Coleoptera | Staphylinidae | | sp.71 | | X | P |
| Insecta | Diptera | Cecidomyiidae | Cecidomyiinae | sp. | | X | P |
| Insecta | Diptera | Ceratopogonidae | | sp. | X | X | P/A |
| Insecta | Diptera | Culicidae | *Culex* | sp. | X | | P/A |
| Insecta | Diptera | Drosophilidae | *Drosophila* | *eleonorae* | X | X | E/P/A |
| Insecta | Diptera | Milichiidae | *Milichiella* | sp. | X | | P |
| Insecta | Diptera | Milichiidae | Milichiinae | sp. | X | X | P |
| Insecta | Diptera | Milichiidae | *Pholeomyia* | sp. | | X | P/A |
| Insecta | Diptera | Muscidae | | jovem | X | X | P/A |
| Insecta | Diptera | Mycetophilidae | *Baeopterogyna* | sp. | | X | P |
| Insecta | Diptera | Phoridae | Phorinae | sp. | | X | P |
| Insecta | Diptera | Psychodidae | *Pericoma* | sp. | X | X | P/A |
| Insecta | Diptera | Psychodidae | *Telmatoscopus* | sp. | X | X | P/A |
| Insecta | Diptera | Streblidae | *Trichobius* | sp. | X | X | P/A |
| Insecta | Hymenoptera | Braconidae | | sp.2 | X | | A |
| Insecta | Hymenoptera | Braconidae | | sp.4 | | X | E/P |
| Insecta | Hymenoptera | Formicidae | *Acromyrmex* | sp.1 | | X | E |
| Insecta | Hymenoptera | Formicidae | *Camponotus* | *atriceps* | | 3 | P |
| Insecta | Hymenoptera | Formicidae | *Crematogaster* | sp.1 | X | | P |
| Insecta | Hymenoptera | Formicidae | *Crematogaster* | sp.3 | | X | P |
| Insecta | Hymenoptera | Formicidae | *Gnamptogenys* | *striatula* | X | X | P |
| Insecta | Hymenoptera | Formicidae | *Pachycondyla* | *striata* | | 1 | P |
| Insecta | Hymenoptera | Formicidae | *Pheidole* | sp.2 | X | | E |
| Insecta | Hymenoptera | Formicidae | *Solenopsis* | sp.1 | X | | P/A |
| Insecta | Hymenoptera | Formicidae | *Solenopsis* | sp.2 | | X | P |
| Insecta | Hymenoptera | Formicidae | *Wasmannia* | sp.1 | X | X | E/P/A |
| Insecta | Lepidoptera | Noctuoidea | | sp.1 | 1 | 2 | E |
| Insecta | Lepidoptera | Tineoidea | | sp.1 | X | X | P/A |
| Insecta | Lepidoptera | Tineoidea | | sp.6 | | X | E/P |
| Insecta | Orthoptera | Phalangopsidae | *Paraclodes* | sp. | 1 | 8 | E |
| Insecta | Orthoptera | Phalangopsidae | *Phalangopsis* | sp. | 600 | 600 | P/A |
| Insecta | Psocoptera | Psyllipsocidae | | jovem | | X | E |
| Insecta | Thysanoptera | Phlaeothripidae | | sp.3 | X | | A |
| Insecta | Thysanoptera | Thripidae | | sp.1 | | X | E |
| Insecta | Thysanura | Nicoletiidae | | sp.1 | X | | E/P |
| Nematomorpha | | | | sp.1 | | 1 | A |
| Amphibia | Anura | Leptodactylidae | *Leptodactylus* | cf. *vastus* | 4 | 2 | P/A |
| Amphibia | Anura | Strabomantidae | *Pristimantis* | cf. *fenestratus* | 13 | 4 | E/P |
| Mammalia | Chiroptera | Emballonuridae | *Peropteryx* | sp. | 2 | 6 | P |
| Mammalia | Chiroptera | Mormoopidae | *Pteronotus* | sp. | 350 | 350 | P |
| Mammalia | Chiroptera | Phyllostomidae | *Anoura* | sp. | 8 | | P |
| Mammalia | Chiroptera | Phyllostomidae | *Carollia* | *perspicillata* | 2700 | 2700 | P |
| Mammalia | Rodentia | Cricetidae | *Rhipidomys* | sp. | 1 | | A |
| Reptilia | Squamata | Colubridae | *Pseustes* | *sulphureus* | | 1 | P |
| Reptilia | Squamata | Gekkonidae | *Thecadactylus* | sp. | | 1 | P |
